# Trends and Predictors of 30-day Readmission Among Patients Hospitalized with Infective Endocarditis in the United States

**DOI:** 10.7759/cureus.4962

**Published:** 2019-06-21

**Authors:** Deepak Kumar Pasupula, Anusha Ganapati Bhat, Sudeep K Siddappa Malleshappa, Amir Lotfi, Mara Slawsky, Sam Buffer, Quinn Pack, Samir Saba

**Affiliations:** 1 Internal Medicine, University of Pittsburgh Medical Center, Pittsburgh, USA; 2 Internal Medicine, Baystate Medical Center, Springfield, USA; 3 Cardiology, Baystate Medical Center, Springfield, USA; 4 Cardiology, University of Pittsburgh Medical Center, Pittsburgh, USA

**Keywords:** infective endocarditis, 30 day readmission, predictors of readmission, elixhauser comorbidity index, united states

## Abstract

Background

The incidence and 30-day readmission rates of patients with infective endocarditis (IE) are not fully determined. We used the United States Nationwide Readmission Database (NRD) to assess national trends and predictors of 30-day readmission.

Methods

We queried the NRD from 2010 to 2014 and identified patients with index hospitalizations primarily for IE. Univariate and multivariate logistic regression analyses were conducted to identify predictors of 30-day readmission.

Results

A total of 48,500 patients (mean age 58 ± 19 years; 38% women; 6.4% died during index hospitalization) were admitted for IE. There was an annual decrease in hospitalization rates by 1.5%. With an exception for 2014, subsequent 30-day readmission rates remained relatively unchanged. All-cause 30-day readmission occurred in 25.4% of patients, 21.8% of which were due to acute or subacute bacterial endocarditis. Leaving against medical advice (odds ratio (OR): 3.46, 95% CI: 3.12 - 3.84; P <0.001), history of drug abuse and a cardiac implantable electronic device in situ (OR: 2.17, 95% CI: 1.53 - 3.08; P <0.001), fungal IE (OR: 1.5, 95% CI: 1.28 - 1.76; P < 0.001), and uninsured patients (OR: 1.39, 95% CI: 1.12 - 1.74, P <0.001) were the strongest independent predictors of 30-day readmission. Readmission cost ($58 million annually) accounted for 14% of the total hospitalization cost.

Conclusions

The annual incidence of IE in the US decreased slightly from 2010 to 2014, but the 30-day readmission rates remained relatively unchanged. Addressing modifiable predictors of readmission may reduce the financial burden of IE on health care.

## Introduction

Infective endocarditis (IE) is a lethal disease, with nearly 25% mortality at six months [1] and 18% within one year of discharge from index hospitalization [2]. The incidence of IE is estimated at approximately 15 cases per 100,000 people in the United States (US) and is believed to be increasing [3]. A study from the Danish registry in 2011 demonstrated high rates of readmission to the hospital (65%) among IE patients, with the majority (85%) readmitted within one year following discharge [2]. Whether these alarming numbers are specific to the Danish population or whether national trends across the United States mirror them remains unsettled. Due to the paucity of large-scale studies, we conducted the present analysis to assess the trends in incidence of IE admissions, the 30-day readmission rates after index hospitalizations, and the predictors and costs associated with readmissions in the United States using the Nationwide Readmissions Database (NRD).

## Materials and methods

Data source

We conducted a retrospective analysis of the NRD from January 1, 2010, to December 31, 2014. The NRD is the largest all-payer annual national database sponsored by the Agency for Healthcare Research and Quality under the Healthcare Cost and Utilization Project (HCUP) [4], representing 49.1% of the total hospitalizations from 22 states in the United States. The NRD collects deidentified annual discharge data by assigning a unique number to every patient in a given year. Therefore, patients can be tracked across various hospitals in the United States using that number. The NRD data is publicly available from HCUP. We accessed and analyzed these data in compliance with the Health Insurance Portability and Accountability Act (HIPAA) of 1996. This protocol was exempted from the institutional review board’s approval.

The NRD provides deidentified patient demographic and clinical variables (e.g., age, sex, race, and comorbidities), variables to assist with a national estimate (discharge weight, stratum used for weighing), and readmission information (verified patient linkage number, length of stay). For each hospitalization, admission diagnoses and procedures are provided using the International Classification of Diseases, Ninth Revision-Clinical Modification (ICD-9-CM) codes for admissions prior to October 1, 2015.

Study population

We identified all IE index admissions between January 1, 2010, and December 31, 2014, using ICD-9-CM codes 421.0, 421.1, 421.9, 036.42, 098.84, 112.81, 115.14, or 115.94 as primary admission diagnosis (DX1 only). The validity of these codes has been previously published [5]. Patients were excluded from the analysis if their age was < 18 years, if they had missing data, or if they were hospitalized in the month of December because the 30-day readmission during the same calendar year could not be ascertained. Out-of-state admissions also had to be excluded since they would not represent readmission in the State Inpatient Database (SID), which is the building block of the NRD. We computed the Elixhauser Comorbidity Index (ECI) [6], ECI readmission score, and ECI mortality scores [7] using the software provided by the HCUP [8].

Description of variables

In the study sample, age was stratified into the following groups: 18 - 54, 55 - 64, 65 - 74, 75 - 84, and > 85 years, and ECI was categorized into four subgroups: 0, 1, 2, and > 3, which has been adopted in previous studies [9]. HCUP identified hospitals as teaching if they had an American Medical Association-approved residency program, if it was a member of the Council of Teaching Hospitals, or if it had full-time equivalent interns and a resident-to-bed ratio of > 0.25. Hospital size was classified into small, medium, and large based upon the number of hospital beds. Length of stay (LOS) was stratified into the following clusters: < 2, 3 - 4, 5 - 6, and > 7 days.

Infective endocarditis etiology and risk factors

The causative organisms of IE were identified using primary or secondary ICD-9-CM codes during the index admission and are described in Table 1. Common microorganisms (Staphylococcus species, Streptococcus species, Gram-negative bacilli, and fungus) were identified as previously described [5]. Risk factors for IE were classified into the following categories: (1) history of drug abuse, (2) presence of a cardiac implantable electronic device (CIED) in situ, (3) presence of a prosthetic valve, or (4) the absence of any of these medical conditions (native valve). The ICD-9-CM codes for these medical conditions have been summarized in Table 2.

**Table 1:** Patient demographics stratified by 30-day readmission a Other payer includes worker’s compensation, CHAMPUS, CHAMPVA, Title V, and other government programs. b Value expressed as median. c p-value = 0.179. CHAMPUS: Civilian Health and Medical Program of the Uniformed Services; CHAMPVA: Civilian Health and Medical Program of the Department of Veterans Affairs

Demographic Variables	Readmitted within 30-days		Not readmitted within 30-days		p-value
Age Cluster					< 0.001
18 - 54 years	5,384	(44%)	15,006	(41%)	
55 - 64 years	2,395	(19%)	7,196	(20%)	
65 - 74 years	2,038	(17%)	6,099	(17%)	
75 - 84 years	1,639	(13%)	5,266	(15%)	
85 - 90 years	0,849	(7%)	2,630	(7%)	
Females	4,899	(40%)	13,714	(38%)	< 0.001
Etiology					
Staphylococcus species	3,962	(32%)	11,209	(31%)	0.011
Streptococcus species	4,300	(35%)	13,753	(38%)	< 0.001
Gram negative bacillus	792	(6%)	1,937	(5%)	< 0.001
Fungus	230	(2%)	454	(1%)	< 0.001
Primary expected payer					< 0.001
Medicare	5,813	(47%)	16,524	(46%)	
Medicaid	2,234	(18%)	5,692	(16%)	
Private	2,689	(22%)	9,188	(25%)	
Self-pay	979	(8%)	2,932	(8%)	
No charge	152	(1%)	373	(1%)	
Other ^a^	405	(3%)	1,385	(4%)	
Non-elective admission	11,329	(92%)	32,699	(90%)	< 0.001
Bed size of hospital					0.381
Small	1,093	(9%)	3,281	(9%)	
Medium	2,668	(22%)	7,643	(21%)	
Large	8,543	(69%)	25,272	(70%)	
Control/ownership of hospital					< 0.001
Government, nonfederal	1,658	(13%)	4,975	(14%)	
Private, non-profit	8,998	(73%)	26,971	(75%)	
Private, invest-own	1,649	(13%)	4,250	(12%)	
Hospital urban-rural designation					< 0.001
Large metropolitan areas with at least 1 million residents	7,261	(59%)	20,367	(56%)	
Small metropolitan areas with less than 1 million residents	4,193	(34%)	13,414	(37%)	
Micropolitan areas	702	(6%)	1,964	(5%)	
Not metropolitan or micropolitan (non-urban residual)	148	(1%)	452	(1%)	
Teaching status of urban hospitals					0.066
Metropolitan non-teaching	3,933	(32%)	11,975	(33%)	
Metropolitan teaching	7,521	(61%)	21,805	(60%)	
Non-metropolitan	850	(7%)	2,416	(7%)	
Disposition					< 0.001
Alive during initial admission	11,300	(92%)	33,146	(92%)	
Post-acute care facility	3,984	(32%)	9,413	(26%)	
Short-term hospital	586	(5%)	1,618	(4%)	^c^
Against medical advice	847	(7%)	817	(2%)	
Died during initial admission	0	(0%)	3,050	(8%)	
Died during readmission	1,004	(8%)	0	(0%)	
Cost of hospitalization during index admission ($)	36,265		38,597		< 0.001
Cost of hospitalization during readmission ($)	23,535				
Length of stay during index admission (days)^ b^	10		10		< 0.004
Length of stay during readmission (days) ^b^	6				

**Table 2:** Etiological agent among infective endocarditis patients ICD-9-CM: International Classification of Diseases, Ninth Revision-Clinical Modification

Microorganism	ICD-9-CM codes during the index admission	
Staphylococcus species		0381, 03810, 03811, 03812, 03819, 0411, 04111, 04112, 04110 and 04119	
Streptococcus species		0380, 0382, 0410, 04100, 04101, 04102, 04103, 04104, 04105, 04109 and 0412	
Gram negative bacillus		0384, 03840, 03841, 03842, 03843, 03844, 03849, 0413, 0414, 0415, 0416, 0417 and 04185	
Fungus		1125, 11281, 1160, 11504, 11514, 11594 and 1173	

Endpoints

The primary endpoint of the study was to assess the annual trends in IE hospitalizations, 30-day readmission rates, and predictors of readmissions. Time to readmission was computed as the difference between index hospitalization discharge and subsequent hospitalization for any cause. If there were multiple readmissions during the 30-day period after the index hospitalization, then only the first readmission was considered. Secondary endpoints included identifying an etiological agent for IE, the top 10 reasons for readmission, and the cost of index and readmission hospitalization. Reasons for readmission were identified using primary diagnosis ICD-9 CM codes for readmission hospitalizations. All ICD-9 CM codes for similar diagnoses were pooled together. The costs associated with the index and readmission hospitalizations were computed in terms of 2014 dollars by using the consumer price index data for medical care released by the United States Department of Labor, Bureau of Labor Statistics [10].

Statistical analysis

Categorical variables are presented as weight values and percentages and compared between patients with versus without readmissions using the χ2 test. Continuous variables are presented as mean ± standard error and compared between patients with versus without readmissions using the Wilcoxon rank test. Multivariable logistic regression for age cluster, sex, ECI, mode of acquisition, etiology, LOS, disposition on index admission, and primary payer were conducted to assess independent predictors for 30-day readmission. A linear regression analysis was performed for continuous variables, as appropriate, to evaluate for the significance of trends. Statistical analyses were performed using IBM SPSS version 26.0 (IBM Corp, Armonk, NY, US).

## Results

**Figure 1:**
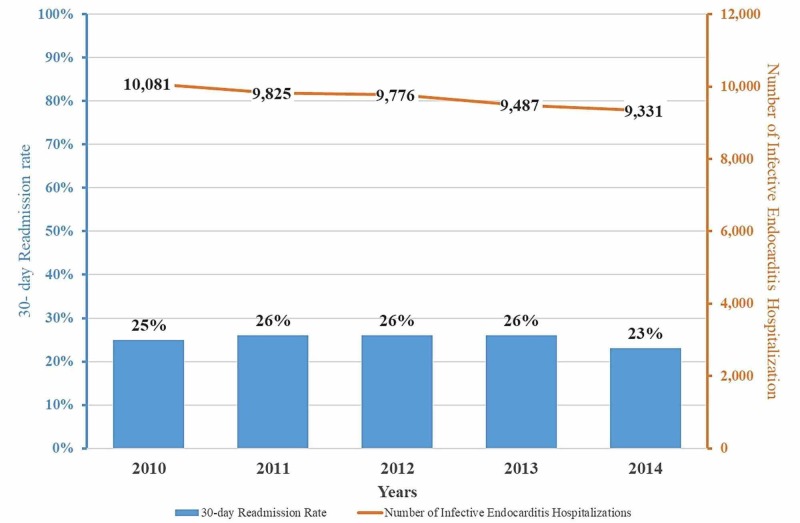
Trends in infective endocarditis hospitalization and all-cause 30-day readmission rates The absolute number of patients hospitalized for infective endocarditis decrease consistently over the study period but all-cause 30-day readmission rates increase slightly until 2013 and then decline in 2014.

The study cohort consisted of 21,076 unique patients admitted with a primary diagnosis of IE between January 1, 2010, and November 30, 2014, representing 48,500 observations, after applying discharge weights. Over the study period, there was a slow but steady decline in the number of IE hospitalizations (10,081 (2010) vs 9,331 (2014); p <0.001) with the incidence decreasing by 1.5% annually. Trends in IE hospitalization and associated 30-day readmissions are shown in Figure 1.

Table 1 details the demographic and clinical characteristics of IE patients as well as the characteristics of their hospital admission. In the study cohort, the largest proportion of patients admitted with IE belonged to the 18-54 years age group (41%). The mean age of the overall cohort was 58 + 19 years, 38% were women, and the majority of patients had significant comorbidities (52% with ECI ≥ 3). Approximately, 46% of the patients had Medicare as their primary insurance provider. Nearly 58% of patients were admitted into a metropolitan (area with ≥ 1 million residents) teaching hospital and nearly two-thirds of the hospitalizations were at large, private (non-profit) organizations. In-hospital mortality among all patients admitted with IE was 6.4%.

30-day readmissions

**Figure 2:**
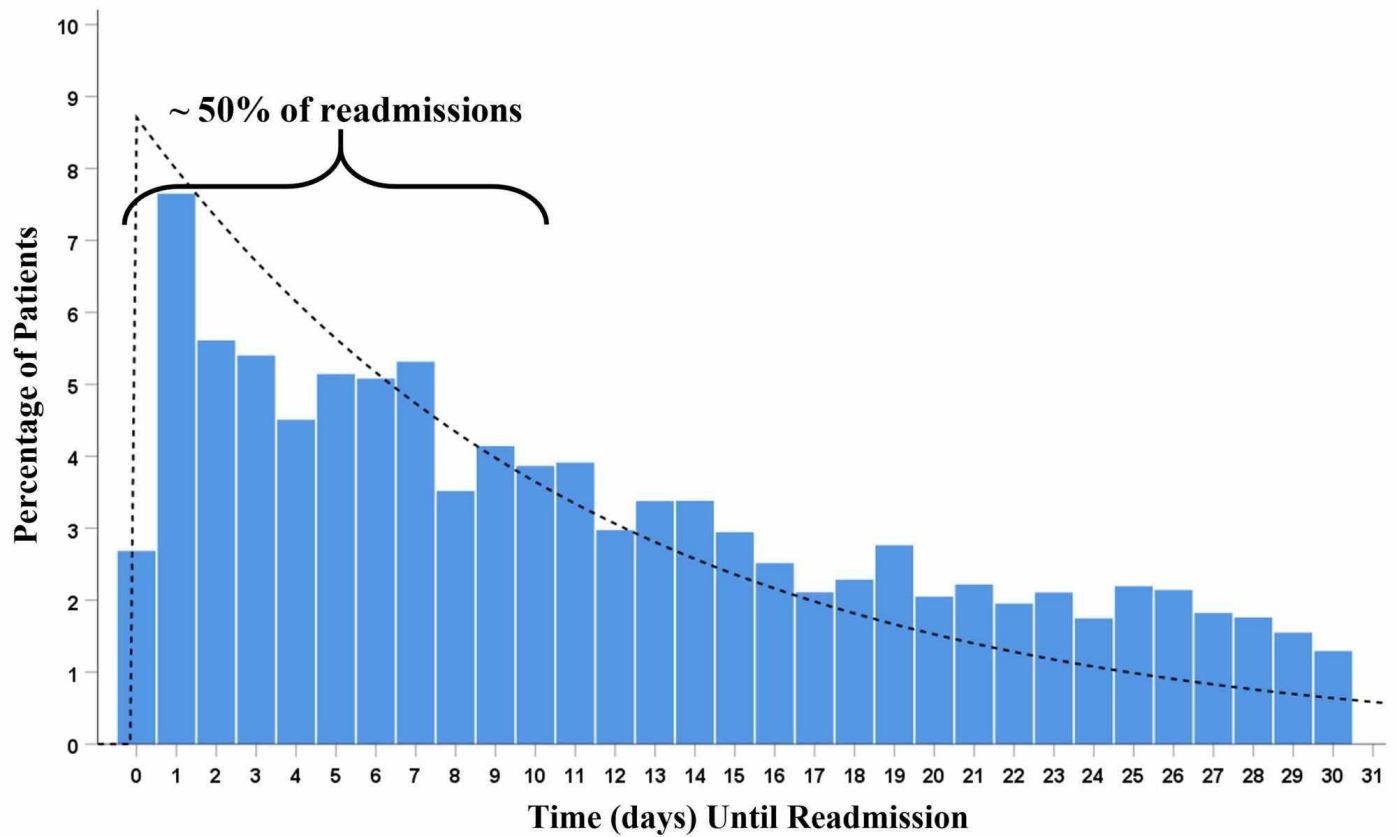
Temporal trend in all-cause 30-day readmission of the total cohort after hospitalization for infective endocarditis (exponential curve histogram) Nearly half of the patient population after being discharged from hospitalization due to infective endocarditis get readmitted in the first 10 days.

Among those who survived the initial hospitalization, the 30-day readmission rate for the total cohort was 25.4%. The annual readmission rate was mostly unchanged from 2010 to 2013 but decreased in 2014 by 11.5% (Figure 1). The time to 30-day readmission after the index IE hospitalization is displayed in Figure 2.

The highest readmission rate of 7.6% was noted within the first 24 hours of discharge. During the index hospitalization, 22.3% of patients had valve surgery as compared to 12.1% during readmission. A small number of patients (n = 64, 0.1%) had valve surgeries during both hospitalizations.

The demographics of patients stratified by 30-day readmission have been summarized in Table 2. When compared to those who did not get readmitted, patients readmitted were more likely to be women (40% vs 38%, p <0.001), between the age group of 18 and 54 years (44% vs 41%, p <0.001), and were admitted emergently (elective admission, 8% vs 10%; p <0.001). Hospitals in larger metropolitan areas (more than one million residents) had higher readmission rates (59% vs 56%, p <0.001) while private non-profit hospitals had less readmissions (73% vs 75%, p<0.001). The readmitted patient had significantly higher ECI (≥ 3), as summarized in Table 3. The average ECI readmission score was significantly higher among patients readmitted within 30 days (15 vs 14, p < 0.001). Although the average ECI mortality score was the same in both the groups, there was a 28% increase in mortality rate among patients who got readmitted within 30 days (6.4% mortality during index admission vs 8.2% mortality during readmission, p <0.001). Among patients who were readmitted, Staphylococcus species, Gram-negative bacilli, and fungal organisms were the most common organisms for IE while Streptococcus species was more common among those who were not readmitted (Table 2).

**Table 3:** Risk factors of infective endocarditis ICD-9-CM: International Classification of Diseases, Ninth Revision-Clinical Modification; CIED: Cardiac implantable electronic device (pacemaker and defibrillator)

History	ICD-9-CM codes during the index admission
Drug Abuse	304, 3040, 3041, 3042, 3043, 3044, 3045, 3046, 3047, 3048, 3049, 30400, 30401, 30402, 30403, 30410, 30411, 30412, 30413, 30420, 30421, 30422, 30423, 30430, 30431, 30432, 30433, 30440, 30441, 30442, 30443, 30450, 30451, 30452, 30453, 30460, 30461, 30462, 30463, 30470, 30471, 30472, 30473, 30480, 30481, 30482, 30483, 30490, 30491, 30492, 30493, 305, 3052, 3053, 3054, 3055, 3056, 3057, 3058, 3059, 30520, 30521, 30522, 30523, 30530, 30531, 30532, 30533, 30540, 30541, 30542, 30543, 30550, 30551, 30552, 30553, 30560, 30561, 30562, 30563, 30570, 30571, 30572, 30573, 30580, 30581, 30582, 30583, 30590, 30591, 30592, 30593, 9650, 96500, 96501, 96502 and 96509
CIED in situ	99672, 99661, V4500, V4501, V4502, V4509, V5331, 99601 and 99604
Prosthetic Valve	V433, V422, 99602 and 99671

The identity of IE organisms remained relatively stable over the study period, except for the presence of Gram-negative bacilli, which decreased by 9.3% annually (Table 4). The association of IE with a history of drug abuse increased by 10.6% annually over the study period while those with native valve endocarditis decreased only by 4.2% annually. These trends have been summarized in Table 4.

**Table 4:** Elixhauser comorbidity index of patients stratified by 30-day readmission ECI: Elixhauser comorbidity index. a Value expressed as mean (standard error)

	Readmitted within 30-days		Not readmitted within 30-days		p-value
Comorbidities					
Congestive heart failure	146	(1%)	474	(1%)	0.294
Valvular disease	111	(1%)	353	(1%)	0.472
Pulmonary circulation disease	166	(1%)	455	(1%)	0.432
Peripheral vascular disease	1,744	(14%)	4,602	(13%)	<0.001
Paralysis	280	(2%)	828	(2%)	0.938
Other neurological disorders	1,082	(9%)	3,042	(8%)	0.181
Chronic pulmonary disease	1,035	(8%)	2,319	(6%)	<0.001
Diabetes without chronic complications	2,317	(19%)	6,439	(18%)	<0.001
Diabetes with chronic complications	1,073	(9%)	2,453	(7%)	<0.001
Hypothyroidism	1,164	(9%)	3,271	(9%)	0.159
Liver disease	1,237	(10%)	3,073	(8%)	<0.001
Peptic ulcer disease with bleeding	10	(0%)	30	(0%)	0.957
Lymphoma	149	(1%)	413	(1%)	0.531
Metastatic cancer	158	(1%)	487	(1%)	0.608
Solid tumor without metastasis	212	(2%)	438	(1%)	<0.001
Rheumatoid arthritis/collagen vascular disorder	408	(3%)	1,247	(3%)	0.495
Coagulopathy	1,876	(15%)	5,718	(16%)	0.147
Obesity	1,290	(10%)	3,414	(9%)	<0.001
Weight loss	1,417	(12%)	4,202	(12%)	0.782
Fluid and electrolyte disorders	4,849	(39%)	13,742	(38%)	0.004
Chronic blood loss anemia	194	(2%)	453	(1%)	0.007
Deficiency anemias	5,432	(44%)	14,363	(40%)	<0.001
Alcohol abuse	667	(5%)	2,028	(6%)	0.447
Drug abuse	2,337	(19%)	6,470	(18%)	0.005
Psychoses	655	(5%)	1,608	(4%)	<0.001
Hypertension	3,461	(28%)	11,150	(31%)	<0.001
Depression	1,576	(13%)	3,715	(10%)	<0.001
Cardiac	11,687	(95%)	34,455	(95%)	0.344
Human immunodeficiency virus	75	(1%)	164	(0%)	0.032
Elixhauser Comorbidity Index					<0.001
0	719	(6%)	2,973	(8%)	
1	1,850	(15%)	6,231	(17%)	
2	2,976	(24%)	8,757	(24%)	
> 3	6,759	(55%)	18,235	(50%)	
ECI Readmission Score ^a^	15 + 0.1		14 + 0.06		<0.001
ECI Mortality Score ^a^	6 + 0.09		6 + 0.05		0.693

The top 10 most common reasons for readmission stratified by year are summarized in Table 5. The most common cause of readmission was acute on subacute bacterial endocarditis (421.0) and the second most common cause was septicemia (038.9). These remained unchanged during the study period. Infection due to a cardiac device, implant, and graft was the next most common reason for readmission (996.61). Heart failure (acute on chronic systolic heart failure (428.23) and acute on chronic diastolic heart failure (428.33)) steadily increased over the study period as causes for readmission after IE hospitalization.

**Table 5:** Trends in etiology, risk factors, and mortality among infective endocarditis patients CIED: Cardiac implantable electronic device (pacemaker and defibrillator)

	2010			2011			2012			2013			2014		
Etiology															
Staphylococcus species	3,157		(31%)	3,172		(32%)	3,067		(31%)	2,813		(30%)	2,963		(32%)
Streptococcus species	3,867		(38%)	3,697		(38%)	3,529		(36%)	3,491		(37%)	3,468		(37%)
Gram negative bacillus	669		(7%)	631		(6%)	497		(5%)	520		(5%)	410		(4%)
Fungus	140		(1%)	151		(2%)	150		(2%)	105		(1%)	138		(1%)
Risk factors															
History of drug abuse	1,222	(12%)		1,359	(14%)		1,656	(17%)		1,789	(19%)		2,018	(22%)	
CIED in situ	547	(5%)		525	(5%)		567	(6%)		499	(5%)		423	(5%)	
History of prosthetic valve	621	(6%)		597	(6%)		666	(7%)		627	(7%)		588	(6%)	
History of drug abuse & CIED in situ	29	(0%)		29	(0%)		28	(0%)		25	(0%)		19	(0%)	
History of drug abuse & history of prosthetic valve	45	(0%)		82	(1%)		81	(1%)		133	(1%)		103	(1%)	
CIED in situ & history of prosthetic valve	230	(2%)		220	(2%)		188	(2%)		220	(2%)		180	(2%)	
History of drug abuse & CIED in situ & history of prosthetic valve	15	(0%)		11	(0%)		36	(0%)		39	(0%)		40	(0%)	
Native valve	7,372	(73%)		7,003	(71%)		6,555	(67%)		6,155	(65%)		5,958	(64%)	
Mortality															
During index hospitalization	734	(7%)		674	(7%)		629	(6%)		513	(5%)		544	(6%)	
During readmission	234	(9%)		234	(9%)		198	(8%)		178	(7%)		159	(7%)	

**Figure 3:**
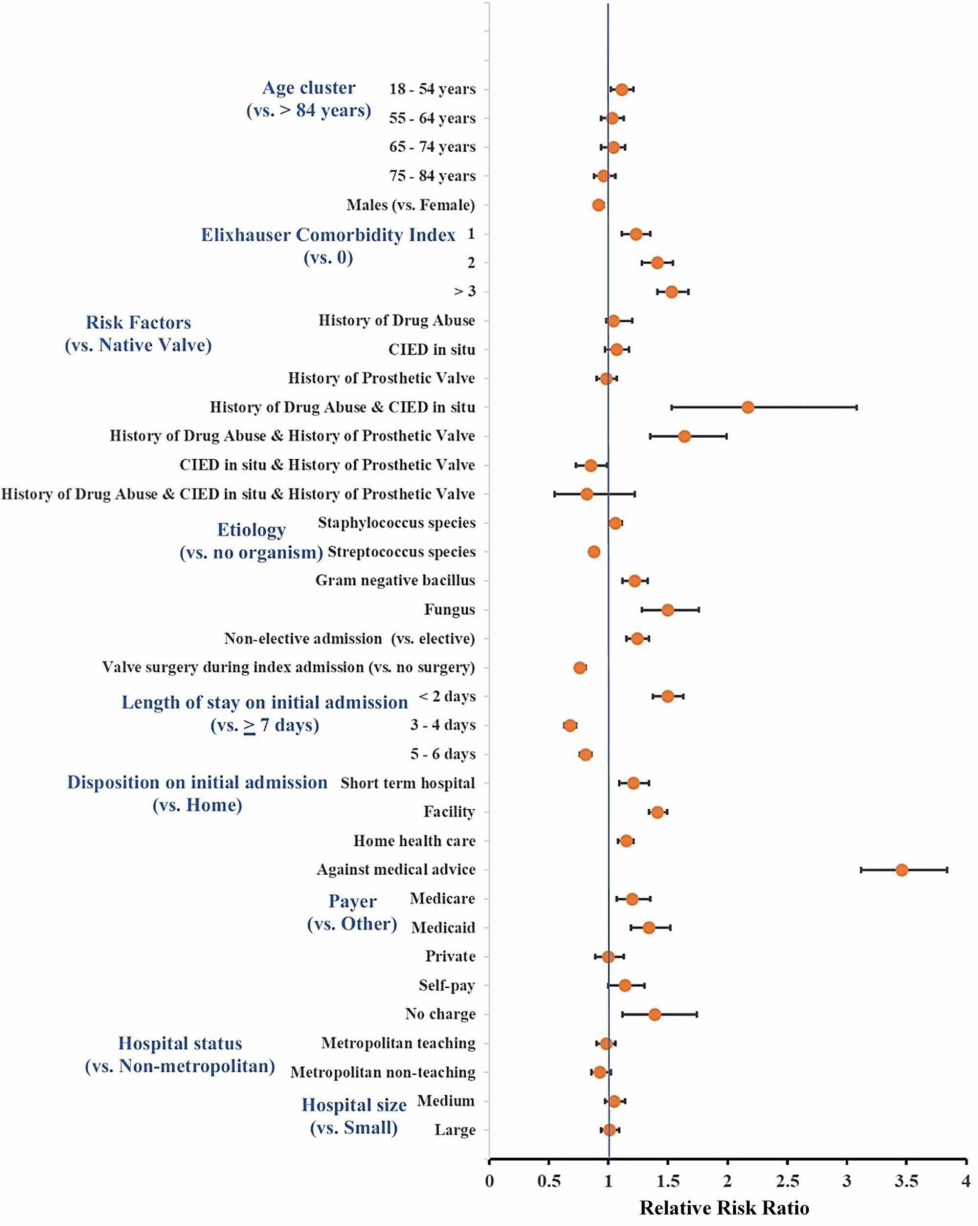
Predictors of 30-day readmission among infective endocarditis patients in the United States The strongest independent predictor for 30-day readmission after hospitalization for infective endocarditis were patients leaving against medical advice, followed by those with a history of drug abuse with a CIED and a history of drug abuse with a prosthetic valve. CIED: cardiac implantable electronic device

In a multivariate regression analysis (Figure 3), the strongest independent predictor for 30-day readmission was leaving the hospital against medical advice (AMA) (relative risk ratio (RR): 3.46, 95% CI: 3.12 - 3.84; p <0.001), followed by patients with a history of drug abuse associated with CIED in situ (RR: 2.17, 95% CI: 1.53 - 3.08; p <0.001), and patients with a history of drug abuse and prosthetic valve (RR: 1.64, 95% CI: 1.35 - 1.99; p <0.001). A higher ECI was also a strong predictor for readmission (RR: 1.53, 95% CI 1.41 - 1.67; p <0.001 for ECI ≥3 and RR: 1.41, 95% CI 1.28 - 1.54; p <0.001 for ECI = 2, both compared to ECI = 0). Compared to patients in whom no organisms could be recovered, patients with fungal IE were most likely to be readmitted (RR: 1.5, 95% CI: 1.28 - 1.76; p < 0.001), followed by those with Gram negative bacillus IE (RR: 1.22, 95% CI: 1.12 - 1.33; p < 0.001). Conversely, patients with Streptococcal IE (RR: 0.88, 95% CI: 0.84 - 0.92; p < 0.001) had lower 30-day readmission rates. Being uninsured (RR: 1.39, 95% CI: 1.12 - 1.74, p <0.001), on Medicare (RR: 1.2, 95% CI: 1.07 - 1.35; p = 0.002), or on Medicaid (RR: 1.34, 95% CI: 1.19 - 1.52; p < 0.001) were also independent predictors for 30-day readmission as compared to other payers.

Cost of hospitalization

**Figure 4:**
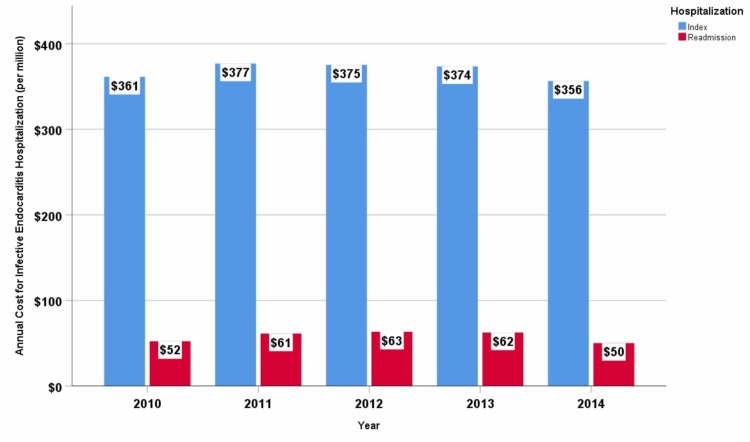
Trends-in-annual-cost-(per-million)-for-index-and-30-day-readmission-hospitalization-for-infective-endocarditis-in-the-United-States Infective endocarditis-related index hospitalization and readmission hospitalization cost gradually increased until 2012 and then declined until 2014. Cost of hospitalization is adjusted for inflation by computing in terms of 2014 dollar for medical care.

During the study period, the average cost per index hospitalization was $38,006, and per readmission, hospitalization cost was $23,535. The annual cost for index hospitalization was approximately $369 million while for readmission hospitalization, it was $58 million. Trends in annual index hospitalization costs and readmission hospitalization costs are summarized in Figure 4. Readmissions accounted for 14% of the total annual cost incurred toward inpatient care. Medicare and Medicaid together incurred $38 million annually towards readmission reimbursement alone, followed by private carriers ($13 million per annum), self-pay ($4 million per annum), other insurance agencies (worker’s compensation, Civilian Health and Medical Program of the Uniformed Services (CHAMPUS), Civilian Health and Medical Program of the Department of Veterans Affairs (CHAMPVA), Title V, and other government programs) ($2 million per annum) and the uninsured ($0.6 million per annum).

## Discussion

In this nationwide analysis of IE from the largest available readmission database in the United States, we report a slight decrease in the admission rates for IE and mostly stable 30-day readmission rates annually (except during 2014) from January 1, 2010, to November 30, 2014. The highest readmission rate was within 24 hours of the index hospitalization and nearly half of the patients were readmitted within 10 days after discharge. Acute and subacute bacterial endocarditis was the most common primary diagnoses upon readmission, followed by septicemia and heart failure. Patients leaving the hospital against medical advice and those with a history of drug use in association with implanted cardiac devices or valve prostheses were at the highest risk for readmission in multivariable analyses. Approximately, $58 million are spent annually in the US toward IE rehospitalization.

Appropriate antibiotic therapy and removal of infected prostheses remain the cornerstone therapy in the management of IE. Practice guidelines are well-developed and regularly updated to assist clinicians in choosing the appropriate treatment for their patients [11-13]. Contrary to findings by Pant et al. [14], who documented an earlier increase in hospitalizations for IE, our study demonstrates a slight decline in the incidence of IE after 2010. This may be a real change in trends or possibly explained by essential differences in methodology whereby we used the NRD as opposed to the nationwide inpatient sample. Also, Pant et al. have used total IE admissions in a given year to estimate the incidence of the disease, which could have overestimated the true incidence by counting the same patient multiple times if they had more than one IE admission in the same year. The NRD has been well-validated in previous studies for its accurate representation of national trends in incidences of disease hospitalizations and for its ability to assess readmission rates [15-16].

IE patients have higher all-cause 30-day readmission rate (25.4%) when compared to the all-cause readmission rate for total inpatient stays in 2014 (13.9% in HCUP statistical brief # 230) [17]. When compared to the top 20 principal diagnoses with the highest seven-day and 30-day readmission rates in 2014, our data in 2014 showed that IE patients have the highest all-cause seven-day (9.4% vs 9.0% (the #1 cause was schizophrenia and other psychotic disorders)) and 30-day readmission rate (23.3% vs 23.2% (the #1 cause was congestive heart failure)). Higher readmission rates among IE patients, when compared to other medical conditions, could not only be associated with higher comorbidities (approximately two-thirds of patients have ECI > 2) but also related to social issues that may lead to suboptimal management and recurring infection. A significant proportion of our IE cohort consisted of patients with a history of drug abuse. This problem, which is reaching epidemic proportions with a 13% increase in the illicit drug abuse from 2002 to 2013 in the US [18], may impede management plans with patients not only exhibiting non-compliance with treatment but also are likely to be associated with continued high-risk behavior leading to recurrent IE. In light of the recent study by Iversen et al., IE can be treated with oral antibiotics in an outpatient setting among few selected group of patients, which may reduce LOS for index and readmission hospitalizations and possibly allow appropriate or near appropriate management of patients leaving AMA [19]. Despite having the highest readmission rate, IE is not among the six conditions/procedures used by Center for Medicare and Medicaid Services as part of the Hospital Readmission Reduction Program (HRRP) to assess the hospital excess readmission ratio, which is used to penalize hospitals with high readmissions rates [20].

Septicemia (038.9) has remained the second most common primary reason for readmission, which most likely stems from uncontrolled and failed initial therapy from IE bacteremia. It is interesting that previous analyses from our group have shown that septicemia is also the second most common cause for readmission after CIED implantation. It is difficult to tease out, based on our study, if bacteremia leads to infection of the CIED or if IE was due to a poor sterile technique, leading to contamination of the pocket site, which caused septicemia. However, in light of our previous study [15] with a very low incidence of cellulitis or abscess of the chest wall (0.04%), we can safely speculate that bacterial seeding caused infection of CIED in situ.

In the multivariate analysis, we demonstrate that patients at higher risk for readmission within 30 days were those who left the hospital AMA, had a history of drug abuse and CIED in situ, had a history of drug abuse and prosthetic valve, having fungal IE, higher comorbidity (ECI > 3), and those with a shorter LOS (< 2 days). Interestingly, our data demonstrate that it is the combination of risk factors (drug abuse and the presence of CIED or prosthetic valve) that is associated with higher rates of readmission, not each risk factor independently. This double hit finding makes physiological sense since the high-risk behavior leads to repetitive inoculation with infectious agents while the presence of a foreign device or prosthesis allows for the seeding of the infectious agent, leading to recurrence of IE even after adequate therapy.

This study’s major advantage is that it is based on mandatory data reporting from all regions of the 22 states participating in the NRD. These collectively represent the real world of IE across the United States. With patient weights provided by the HCUP, this dataset provides accurate estimates of IE admissions, readmissions rate, predictors of readmissions, and associated costs.

Limitations

However, our study is limited due to its retrospective nature and its reliance on administrative data. We are unable to validate the accuracy of ICD-9-CM code documentation, which was used to determine the clinical and procedural information pertaining to the disease of interest, however, these errors are expected to be very low [21]. NRD lacks data with regards to ethnicity, socioeconomic status, and clinical data, such as echocardiographic findings of an abscess, perivalvular leaks, presence and size of vegetations, and valvular damage, to mention a few. There is also a lack of data with regards to blood cultures, antibiotic therapy duration after discharge, or compliance with medications. The microorganisms identified using ICD-9-CM codes are assumed to be the causative organisms, however, we are unable to validate these data.

## Conclusions

In the United States, there is a slight decrease in the incidence of IE hospitalizations between January 1, 2010, and November 30, 2014. One in four IE patients is readmitted to the hospital within 30 days. IE patients leaving AMA or having a history of drug abuse and CIED in-situ or prosthetic valve in-situ were at the highest risk for 30-day readmission. The annual cost of readmissions after IE is $58 million. Further research is needed to investigate the impact of clinical and social measures that could decrease patients’ readmissions after IE and curb the high associated costs.
